# Tough and Functional Cross-linked Bioplastics from Sheep Wool Keratin

**DOI:** 10.1038/s41598-019-51393-5

**Published:** 2019-10-15

**Authors:** Borja Fernández-d’Arlas

**Affiliations:** Institute for Advanced Materials (INAMAT), Universidad Pública de Navarra (UPNA), Centro Jerónimo de Ayanz, Pamplona, España Spain

**Keywords:** Mechanical properties, Organic molecules in materials science

## Abstract

Novel bioplastic films derived from wool keratins were prepared by protein solution in an alkaline mild oxidative method that splits disulphide (-S-S-) bonds. The native structure of the keratin macromolecules was partially modified upon extraction as revealed by the decrease of the β-sheet to α-helices/coils ratio but high molecular weight fractions (31, 22 and 13 KDa) was retained permitting film formation and plastic behaviour of films. Keratin films were plasticised with glycerol and sodium dodecyl sulphonic acid (SDS), which provided different hydrophobic character to bioplastics. Water content in the films depend on the relative humidity (RH), being able to absorb up to 35 wt% H_2_O at an ambient of 80% RH. Films were mechanically, thermally and optically analysed. The spectroscopic analyses revelled that these bioplastic films absorb UV light, what is interesting for packaging applications. Thermogravimetric and thermomechanical analysis revealed high stability of keratin macromolecules up to 200 °C with no inherent thermal transitions. Tough bioplastics (19 ± 4 MJ∙ m^−3^) were obtained after thermal cross-linking with glycerol and formaldehyde outperforming mechanical properties previously reported for protein films.

## Introduction

The advances in petrochemical sciences during the 21^th^ century promoted a brand new scientific field known as Polymer Science. This shed the world with a big family of new materials: plastics. Aside the advantageous and unique properties of plastics, they also endow a big CO_2_ finger print associated to their production, transportation and management^1^. In addition, once their life cycle ends they are usually incinerated, what causes more release of CO_2_, or discarded into landfills, if not into seas, where they will stay for decades or hundreds of years^1^. Their use will keep on growing exponentially^1^ and it will be also the task of polymer scientists to amend the problems arising from plastic production and disposal. Transition from a Fossil to a Circular Economy encompasses with the development of sustainable plastics. One attempt to tackle this situation is the substitution of oil based plastics by compostable bioplastic constituted by biomacromolecules derived from plants, like the case of bioplastics based on potato or corn starch^2,3^. Other natural biopolymers being studied as possible candidates for bioplastics include polysaccharides like chitin from shellfish^4^, alginates from seaweeds^5,6^, cellulose^7^, or proteins derived from soy^8–10^, sunflower^11^, milk^12–14^, whey^15^, or feather^16^ and fish^17^ residues. These last two examples are attempts for a Circular Economy, in which residues are provided with a value^18,19^.

Bioplastics from plants have the advantage of adsorbing CO_2_ during the photosynthesis. In addition, after their life cycle they can be easily integrated into the environment since they are usually biodegradable. In principle, these two factors might minimize their energy consumption and CO_2_ fingerprint in comparison to common *fossil*-plastics^1^. But, the problem of certain bioplastics, like the ones derived from plants is that primarily human resources might be deviated for bioplastic production. This situation would be similar to that of plant derived biodiesels^20,21^ leading to a rise in food prices and motivating deforestation. It is for these reasons that prime mater for future bioplastics should be search into residues from currently human activities. This concept would allow a better management of these residues and would mitigate the problem associated to plastics end of life cycle. In certain countries wool has been rendered with no outcome due to the impossibility to compete with “tailor-made” synthetic fibers. The management of these residues constitute a serious challenge and authorities have already pointed the need of finding an outcome for them^22,23^. Following up, more recently, a directorate for a Sustainable Bioeconomy has been published, where it is highlighted the importance of developing bioplastics for a Circular Bioeconomy^24^.

A small portion of keratin oligopeptides hydrolysates has been used as cosmetic for humans and low value nurturing additives for pets^25^. These hydrolysates are also being considered as a nitrogen source in the form of amino acids and oligopeptides for structuring the land soil^26,27^. Here we explore the use of keratin derived macromolecules for preparing sustainable biodegradable bioplastics. Wools are up to a 90% of weight composed of keratin fibres, fibrous proteins characterised by the high presence of cystine (*R*_1_-S-S-*R*_2_) residues^28,29^. These act as cross-linking points that provide stiffness and strength to the fibres but also render the fibroins difficult to be extracted by dissolution in common solvents^30^. As in comparison to other proteins like silk, keratins are not dissolved in water solutions of concentrated salts like LiBr, since the *R*_1_-S-S-*R*_2_ cross-links keep the macromolecules non soluble^31^. Therefore, keratin extraction and dissolution requires a reactive extraction in which disulphides are split apart. Other attempts for keratin extraction were based on traditional lab-scale methods for protein denaturalization with Sulphur with reducing agents like mercaptoethanol (HS-CH_2_-CH_2_-OH) or thioglycolic acid (HS-CH_2_-COOH), and high concentration of hydrogen bonding disrupting agents such as urea, O = C(NH_2_)_2_, and thiourea^32^, S = C(NH_2_)_2_. More recent attempts include the use cysteine^33,34^ or ionic liquids^35^ for keratin extraction. The method used here is based on our previous advances in mild oxidative methodologies in which keratin derivatives are obtained in high yields, easily purified and no toxic reactants and residues are used or generated^36^. The keratins derivatives were analysed and its water solutions were used to prepare tough plasticised and cross-linked films that were characterised morphologically, optically and analysed by thermal and mechanical means.

## Results and Discussion

### Keratin extraction and characterization

**Figure 1:**
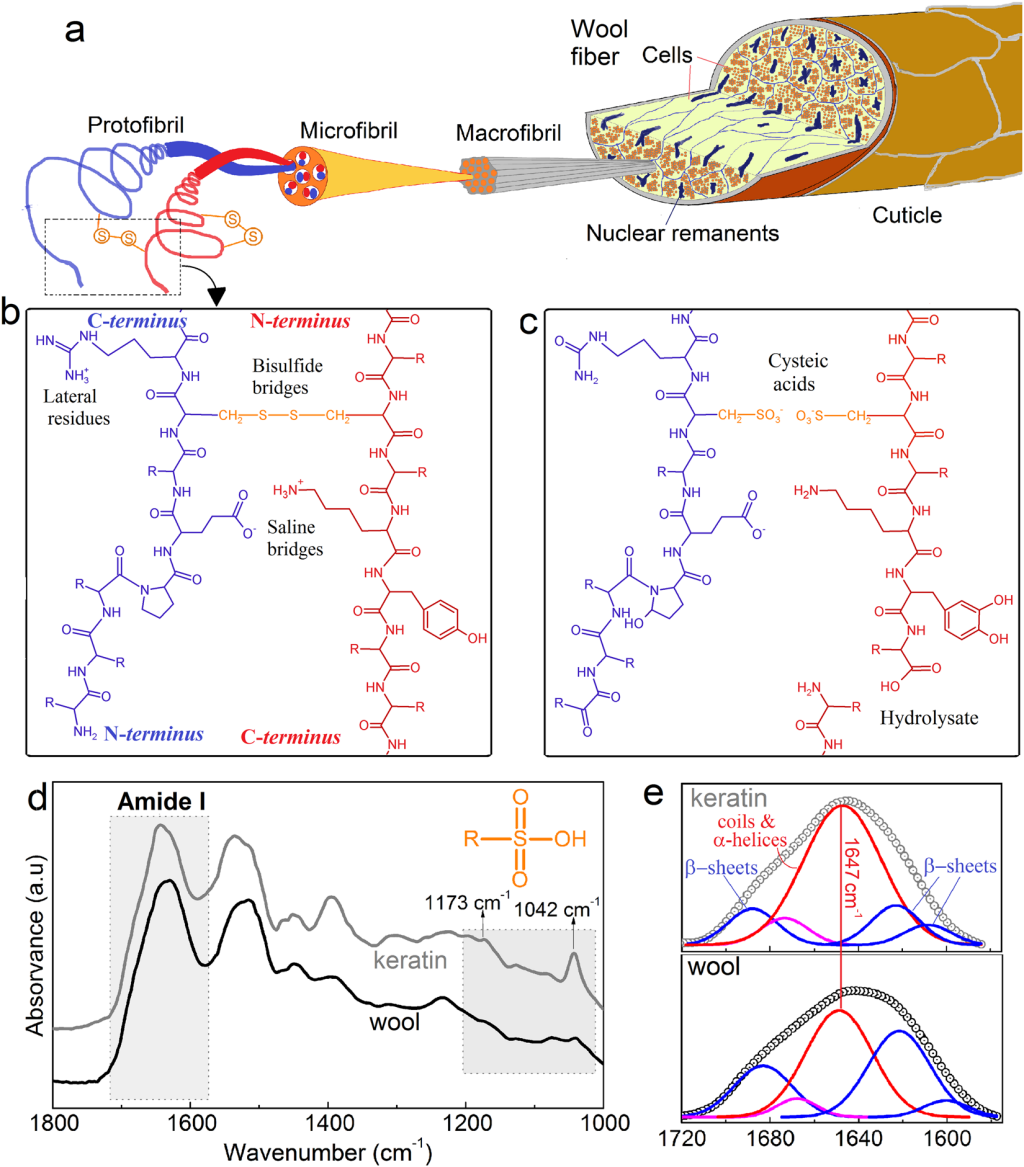
(**a**) Schematic of a wool fibre structure indicating coiled keratin coils rearrangements. (**b**) Schematic of intermolecular interactions within keratin macromolecules. (**c**) Keratin macromolecules after oxidation treatment. (**d**) FTIR spectra in the range 1800–1000 cm^−1^ of wool and extracted keratin film indicating the amide I and sulphoxide absorptions. (**e**) Deconvoluted amide I region (1700–1600 cm^−1^) of wool and extracted keratin.

The morphology of wool has different structural orders and elements, as depicted in Fig. 1a. Keratin macromolecules are entangled in coiled-coils like protofibrils that form microfibrils bundled into bunches of macrofibrils within the cells and associated trough different intermolecular interactions, as covalent bisulfide bridges, ionic interaction or hydrogen bonds (Fig. 1b)^37^. The alkaline oxidation of wool applied here modifies this range of interaction promoting macromolecular disentanglement and separation and proteins dissolution. Extraction reaction of keratin from wool was stopped after 2 h, when almost all of the wool was dissolved into the extractive liquor, as can be seen in Fig. S1a. The mixture was filtered and yield was estimated as 90% of solubilised protein, as determined gravimetrically. Titration of the filtrated (Fig. S1b) shows that a dramatic drop in pH occurs before keratin precipitation at pH = 4.4. This can be related to the protonation of basic amino acids and the approach to the isoelectric point of the oxidised keratin macromolecules. As shown before, keratins obtained by reduction with thioglicolic acid exhibit higher isoelectric points. As it was proposed, the introduction of sulphenic (*R*-SOH), sulphinic (*R*-SO_2_H) or sulphonic (*R*-SO_3_H) acid groups^38^ might be the reason for the drop in the isoelectric point, macromolecular disentanglement and dissolution. In particular, it has been proposed^26^ that the reaction taking place would be:

1 $$K \mbox{-} S \mbox{-} S \mbox{-} K+6{{\rm{H}}}_{2}{{\rm{O}}}_{2}=2K{{ \mbox{-} \mathrm{SO}}_{3}}^{-}+6{{\rm{H}}}_{2}{\rm{O}}$$

where *K*, stands for the keratin macromolecular backbone. With this extraction strategy the main by products are water, oxygen and slight amounts of hydroxides^26^. No toxic by-products are generated and it is minimized the water consumption for washing or dialysis purification processes typical of other keratin extraction routes^39,40^. At pH < 4.4 the keratin derivative precipitated and was collected as a withe precipitate polymer (Fig. S1c). As can be seen in Table 1, elemental analysis of the extracted keratin reveals that upon the oxidative extractive process oxygen is incorporated in a larger proportion to what is expected by Eq. (1). This can be explained by the potential towards oxidation of amino acids such as arginine^41^, tyrosine^42^, and amino terminals^43^, fact that is also depicted in Fig. 1b,c.

**Table 1:** Elemental analysis and secondary structures distribution as analysed by FTIR Amide I (1700–1600 cm^−1^) deconvolution.

Sample	Elemental analysis (mol%)					Secondary structure (FTIR) (area%)		
	C	N	H	S	O	β-sheets	β -turns	α-helices&coils
Wool	30	8	48	1	13	54	4	42
Keratin	27	8	45	1	19	28	7	65

SDS-PAGE electropherograms (Fig. S2) of wool keratins extracted with the reductive method and oxidative methods indicate that extraction trough the reductive method exhibits in the low density gel of 10% Acril/Bis two distinct bands of 61 and 49 KDa which can be related to the typical Type I (acidic) and Type II (basic) bands attributed to mammals keratins^44^, as wools from other breeds^28,29^, or human hair^45^. These bands are not detectable in keratins obtained by the oxidation method employed here for bioplastic preparation (Fig. S2a,b) due to particle hydrolysis of keratin macromolecules trough peptide bond cleavage. Yet other relative high molecular weight fractions with maximum intensities at 31, 22 and 13 KDa were detected in the denser 18% Acril/Bis gel fraction (Fig. S2a,c).

These results are in accordance with previously reported data obtained with other gel configurations^36^. Therefore, this allow to conclude that although a relatively harsh oxidative method is employed, under the adequate conditions reported here, relative high enough molecular weight (13–31 KDa) keratin derived macromolecules were obtained for bioplastic development. This is optimist since this process can be considered to comply with Green Chemistry principles and because the chemicals employed can be derived upon renewable and non-fossil resources.

A recent analysis on the keratin extraction methodologies also revealed that mild oxidative methods, such as treatments with percarbonate (PCC) or paracetic acid (PAA), are economically attractive and yield keratins with relatively high molecular weights^46^. Another feature of the keratins obtained from the oxidative process is that they are more water soluble than keratins obtained by the reductive method as the bisulfide bridges and secondary structures do not trend to re-form^36^. In the reductive process the reformation of *K*-S-S-*K* bridges render the polypeptides thermodynamically incompatible with water, as determined previously by SDS-PAGE, solubility tests^36^ and by static light scattering^47^.

### Macromolecular characterisation of wool and films by FTIR

Wool keratin bioplastic films were analysed by Fourier transformed infrared spectroscopy (FTIR) and the spectra compared to that of native wool. Figure 1d shows the FTIR spectra of a keratin films along that of native wool in the region of 1800–1000 cm^−1^. Common features observed correspond to those typical from proteins^48,49^ such as C = O stretching at 1600–1700 cm^−1^ (Amide I), N-H bending at 1480–1575 cm^−1^ (Amide II), C-N stretching in the range 1230–1300 cm^−1^ (Amide III) and the Amide A band, centred in 3268 cm^−1^, related to N-H stretching. In addition, the spectral region between 1200–1000 cm^−1^, sensitive to the presence of sulphur oxidized derivatives, presents modification upon the oxidative extraction method as observed by comparing the wool and keratin spectra. The keratin films present absorption peaks at 1042 and 1173 cm^−1^. In the immediacy of 1090 and 1045 cm^−1^ bands associated to S = O stretching in sulphinic acids (R-SO_2_H) and their salts have been reported^50,51^. Other authors have also report that the oxidation of wool^52–54^ leads to the increase of peaks at ~1045 cm^−1^ and 1173 cm^−1^, attributed to cysteic acid (K-SO_3_H). Therefore, the increase in intensities relative to the band at 1261 cm^−1^ (related to Amide III mode) of wide bands with peaks at 1173 and 1042 cm^−1^ observed for films in Fig. 1d can be related to the introduction of oxidized sulphur derivatives such as sulphinic or sulfonic (R-SO_3_H) acids.

The analysis of the Amide I (1600–1700 cm^−1^) region, sensitive to the secondary structure of proteins, also reveal important modification upon extraction method. As can be seen in Fig. 1b, the band centred at 1647cm^−1^, associated to amorphous coils and α-helices, increases upon extraction. Data extracted from Amide I deconvolution is gathered in Table 1. The portion of β-sheets, random coils/α-helices and β-turns changes from 54, 42 and 4%, respectively, in the case of wool, to 28, 65 and 7%, in the case of extracted keratin. In particular the β-sheet to coils/helices ratio goes from 1.3 to 0.4 for wool to keratin films, respectively.

### Bioplastic films from wool keratin

**Figure 2:**
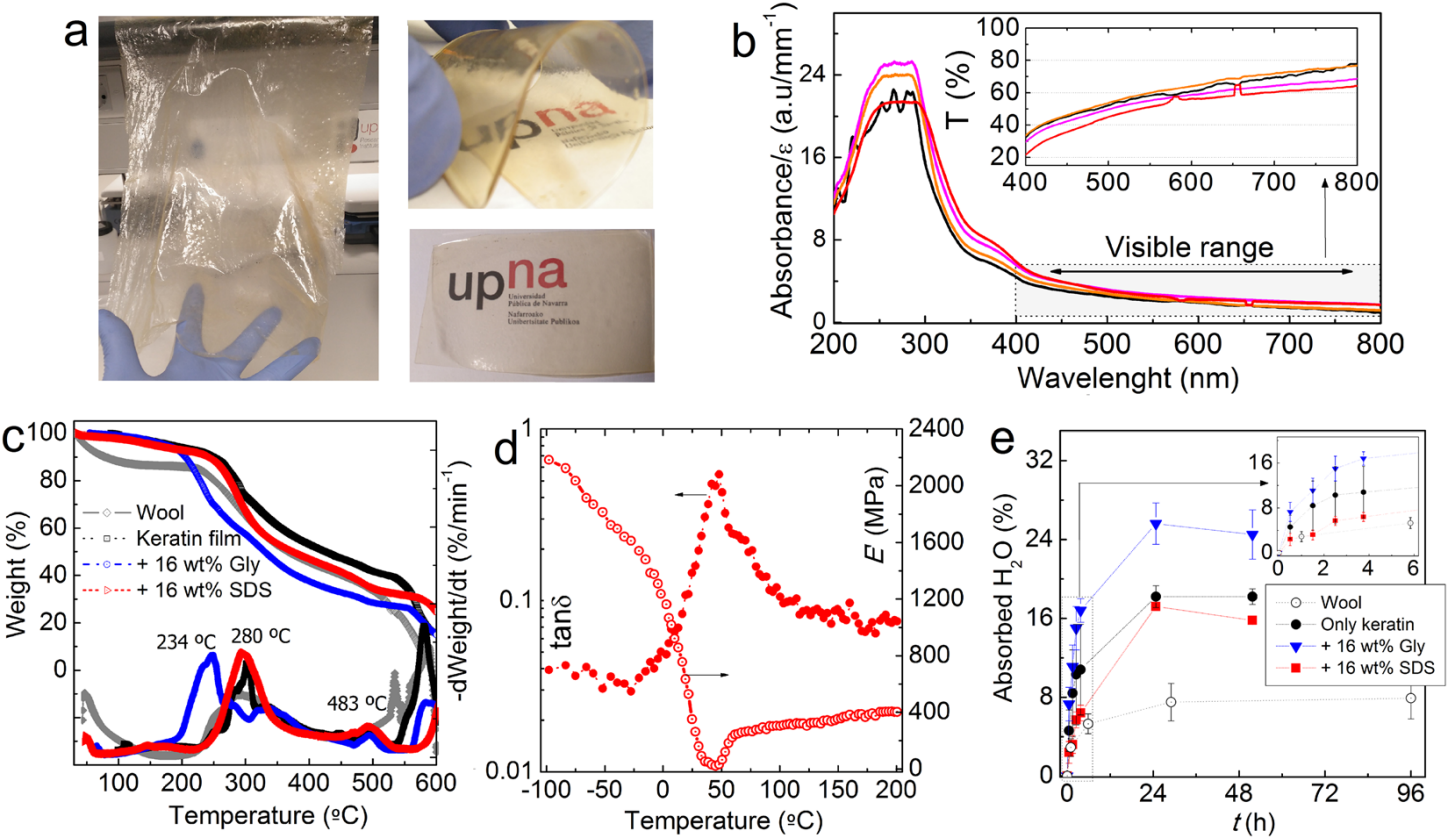
(**a**) Wool keratin films obtained by solvent (H_2_O/isopropanol mixture) casting. The big film was plasticized with sunflower oil and oleic acid whereas the films to the right with 28 wt% glycerol. (**b**) UV-Vis spectra of selected wool keratin films plasticized with different amounts of glycerol. The inset shows the corresponding transmittance spectra in the visible range. (**c**) Thermogravimetric (TGA) and derivative (DTGA) curves of selected wool keratin materials. (**d**) DMTA curves for the storage modulus (E′) and loss tangent (tanδ) of a pure keratin film. (**e**) Water absorption isotherms at 65%RH and 24 ± 3 °C for wool, an extracted keratin films and derived bioplastics with glycerol and SDS.

Films with different plasticizers were prepared by solution casting into silicon moulds. As can be seen in Fig. 2a, the extracted keratin derivative exhibited excellent film forming ability under ambient conditions. The films have a high degree of transparency and were slightly yellowish, presumably due to the presence of amino acids such as tryptophan (6 wt%), histidine (1 wt%), arginine (10 wt%) and proline (5 wt%)^29^. This tendency to exhibit yellowish or brownish colours has been previously reported for the case of other proteins films such as those based on human air keratin^45^, chicken feather keratin^35^, soy proteins^55^, or fish gelatine^56^.

Also, as previous reports in literature for films from many different protein sources^8,16,17,35,45,57,58^ and for other biopolymers^59^ the pure keratin films produced here trend to be brittle under normal ambient conditions (RH <70%) and therefore different amount of glycerol as well as other plasticizers were used in order to modify the mechanical behaviour of films. With this approach materials with a different range of flexibility, stiffness, plasticity and strength could be obtained, as will be reported below.

### Optical properties and UV absorption

Analysis of the films by UV-Vis spectroscopy (Fig. 2b) showed that wool keratins have a high absorbance in the UV range (<390 nm). The absorption of proteins in the UV spectra is related to the presence of conjugate species susceptible to electronic resonance, such as peptide bonds (200–250 nm), tryptophan and histidines, or aromatic amino acids as phenylalanine and tyrosine (200–300 nm) present in wool^29^. As can be extracted by comparing data gathered in Table 2, wool keratin films present a relatively low opacity (i.e., high transparency) as compared to other protein films prepared by casting such as fish gelatine of soy protein films, or other oil derived synthetic plastics such as low density polyethylene (LDPE). The high absorption in the UV range combined with a relatively high transparency in the visible spectra is a very interesting property for packaging, since the embodied materials are protected from the solar UV radiation at the same time they are visible for the user.

**Table 2:** Opacity of wool keratin films as compared to those of other protein and oil derived plastics.

Protein	Opacity (A_600_/mm)	Ref.
Wool keratin (casting)	1.93	This work
Soy protein (compression)	1.38	^55^
Soy protein (compression)	1.16	^61^
Soy protein (casting)	8.71	
Pea protein (casting)	1.5	^70^
Pea protein (casting)	5.6	^71^
Fish gelatine (casting)	6–36*	^72^
OPP	1.67	
LDPE	3.05	
PE	1.51	
PVDC	4.58	

Note:*Function of pH.

### Thermal behaviour

Thermogravimetrical analysis (Fig. 2c) with a set of materials were performed in order to understand the composition and keratin modification impact in films stability. Washes wool fibroins present a maximum in degradation rate at around 280 °C, similar to pure keratin and 16 wt% SDS containing films.

These processes can be related to the beginning of protein degradation, as has also been reported for keratin extracted from Merino wool^28,29^. The film containing glycerol presents a lower degradation process starting at ~180 °C and with a peak at 234 °C, what can be related to the evaporation of glycerol (*T*_b_ = 290 °C) and a higher process with a maximum degradation temperature at 320 °C. A very similar degradation pattern has been previously observed for glycerol plasticized soy protein plastics^60^.

Thermo-mechanical analysis of a keratin film (Fig. 2d) showed the dramatic influence of water plasticizing effect on the mechanical properties of keratin bioplastics (film with ~10 wt% H_2_O). The elastic modulus of the film decreases continuously as the temperature rises from −100 °C and just around 0 °C, when water molecules gain mobility, the modulus drops dramatically almost one decade. In addition, and immediate increase in the modulus was observed around 45 °C, after what the modulus increase was more moderate. This two observations have also been previously reported for soy protein films containing keratin^61^ and were attributed to reformation of chemical cross-links between protein macromolecules.

Another plausible explanation would be the restoration of physical intermolecular associations between keratin macromolecules upon regaining mobility upon water melting. The moderate increase in modulus after 50 °C could be related to the loss of water plasticizing effect due to evaporation and degradation reactions leading to cross-linking.

### Films chemical impact on ambient water absorption

Given the importance of the effect of the presence of water in the bulk of different biopolymer based materials such as protein based films, here we performed a kinetic analysis of water absorption (*T* = 24 ± 3 °C; RH = 65 ± 3%) into wool, as extracted keratin and derived bioplastic films compounded with either glycerol or SDS (Fig. 2e). Table 3 gathers water kinetics constants obtained from analysis of data shown in Fig. 2e.

**Table 3:** Water absorption parameters for different films compared with those of washed wool.

Material	^§^H_2_O absorption rate (%·h^−1^)	^δ^H_2_O at eq. (%)
Wool	1.0 ± 0.2*	7.9 ± 2.1^†^
Keratin	3.6 ± 0.5*	18.2 ± 0.8^†^
Keratin + 16 wt%SDS	1.9 ± 0.3*	15.8 ± 0.3^†^
Keratin + 16 wt%Gly	5.3 ± 0.7*	24.5 ± 2.5^†^

Notes: ^§^Slope of linear fit with data <6 h. ^δ^From last measured data (i.e., at equilibrium).*Linear regression error; ^†^Standard deviation from a set of 3 samples.

It can be seen that pure keratin film already exhibits significant differences with respect to the original wool fibroins. Wool fibres absorb less water albeit their higher porosity as compared to the films. The higher hydrophilic character of films can be associated to the chemical modifications arising from the extractive procedure which introduces more hydrophilic groups such as sulphur oxidative species, as describe in Eq. (1).

Results also cast significant differences depending of the chemical modification introduced to the bioplastic. Pure keratin film exhibits an intermediate behaviour in comparison with films with glycerol and SDS.

The lower rate and water absorbed by films modified with SDS can be related to the amphiphilic nature of this additive. The SDS hydrophilic head (-SO_4_^−^) might be interacting with ionic groups of the keratin macromolecules such as quaternary ammonium groups from histidines, lysines and arginines^62^. On the other hand the hydrophobic tails comprised of a hydrocarbon chain might reduce the hydrophilic nature of the complex by avoiding H_2_O penetration. The opposite situation occurs when hydrophilic glycerol is used as an additive, and higher proportion of H_2_O is absorbed than pure keratin film. These results indicate that with the addition of small proportions of additives (i.e. <20 wt%) is possible to significantly tune the hygroscopic behaviour of the bioplastic. The addition of SDS resulted in ca. 40% less water absorbed in comparison with the film with glycerol. As it is discussed below, water molecules act as a plasticizers. Therefore, the use of amphiphilic additives such as SDS, would tune water absorption and allow controlling the bioplastic mechanical properties.

### Relative humidity influence on mechanical properties

The presence of H_2_O in the ambient (i.e., relative humidity, RH) has a dramatic influence on the water content in the keratin bioplastics, as shown in Table 4. By modifying the RH from 16 to 65% the amount of water within the bioplastic goes from 4.3 ± 0.4 to 33.7 ± 0.6%H_2_O, what has a direct influence in the strength and strain at break of bioplastics, as also shown in Table 4. The strength varies inversely proportional to water content decreasing more than an order of magnitude from 17.9 ± 2.1 MPa at 16% RH to 0.6 ± 0.2 MPa at 65% RH. The strain at break increases by increasing the water content up to a 15% H_2_O but with higher H_2_O contents the strain decreases again, probably due to the lack in cohesion within the material.

**Table 4:** H_2_O content at equilibrium in a bioplastic with 14 wt% glycerol at different relative humidity, and its impact on strength and strain at break.

RH (%)	H_2_O (%)	*ε*_max_ (%)	*σ*_max_ (MPa)
16 ± 2	4.3 ± 0.4	19 ± 6	17.9 ± 2.1
30 ± 2	6.9 ± 0.3	46 ± 11	12.4 ± 2.6
53 ± 2	15.4 ± 0.8	302 ± 44	1.1 ± 0.1
65 ± 3	33.7 ± 0.6	189 ± 53	0.6 ± 0.2

### Plasticized keratin films

**Figure 3:**
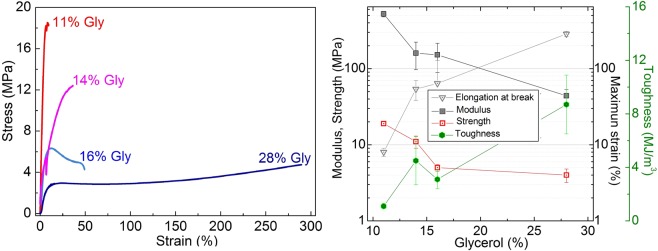
(**a**) Representative stress-strain curves of wool keratin bioplastics plasticized with different amount of glycerol. (**b**) Dependence of elastic modulus, strength, elongation at break and toughness of bioplastics as function of glycerol content.

Keratin was combined with either glycerol or SDS up to a concentration of 28 wt%. In the case of SDS the resulting bioplastics were virtually as brittle as the pure keratin films and could not be tested. In Fig. 3a representative stress-strain curves of bioplastics with different glycerol content are shown. It can be seen the high impact of plasticizer content into mechanical properties. With a glycerol content of 11 wt% the bioplastics behaves relatively as a brittle material, with a small breaking strain high elastic modulus and relatively high breaking stress. By slightly increasing the glycerol content over 16 wt% the material properties were modified substantially (Fig. 3b), increasing the breaking strain up to 50% and decreasing the breaking strength down to 5 MPa. In addition, the material plasticized with more than 16 wt% glycerol presented a typical stress-strain behaviour of a plastic with a yield at about 10% deformation and 6 MPa of stress, followed by a plastic deformation up to failure. The toughness of bioplastics trend to increase with glycerol content. The brittle nature of non-plasticized keratin films in comparison to wool fibres, which are typically strong (150–200 MPa), compliant (extensibility = 30–60%) and therefore relatively tough fibres^63^, can be related to the chemical and morphological modifications exposed above, as determined by FTIR spectroscopy and SDS-PAGE. Splitting of bisulfide bridges, disruption of β-sheets and partial hydrolysis of the macromolecules reduce mechanical stress transfer capability and decrease fracture toughness.

### Cross-linking of keratin bioplastics

**Figure 4:**
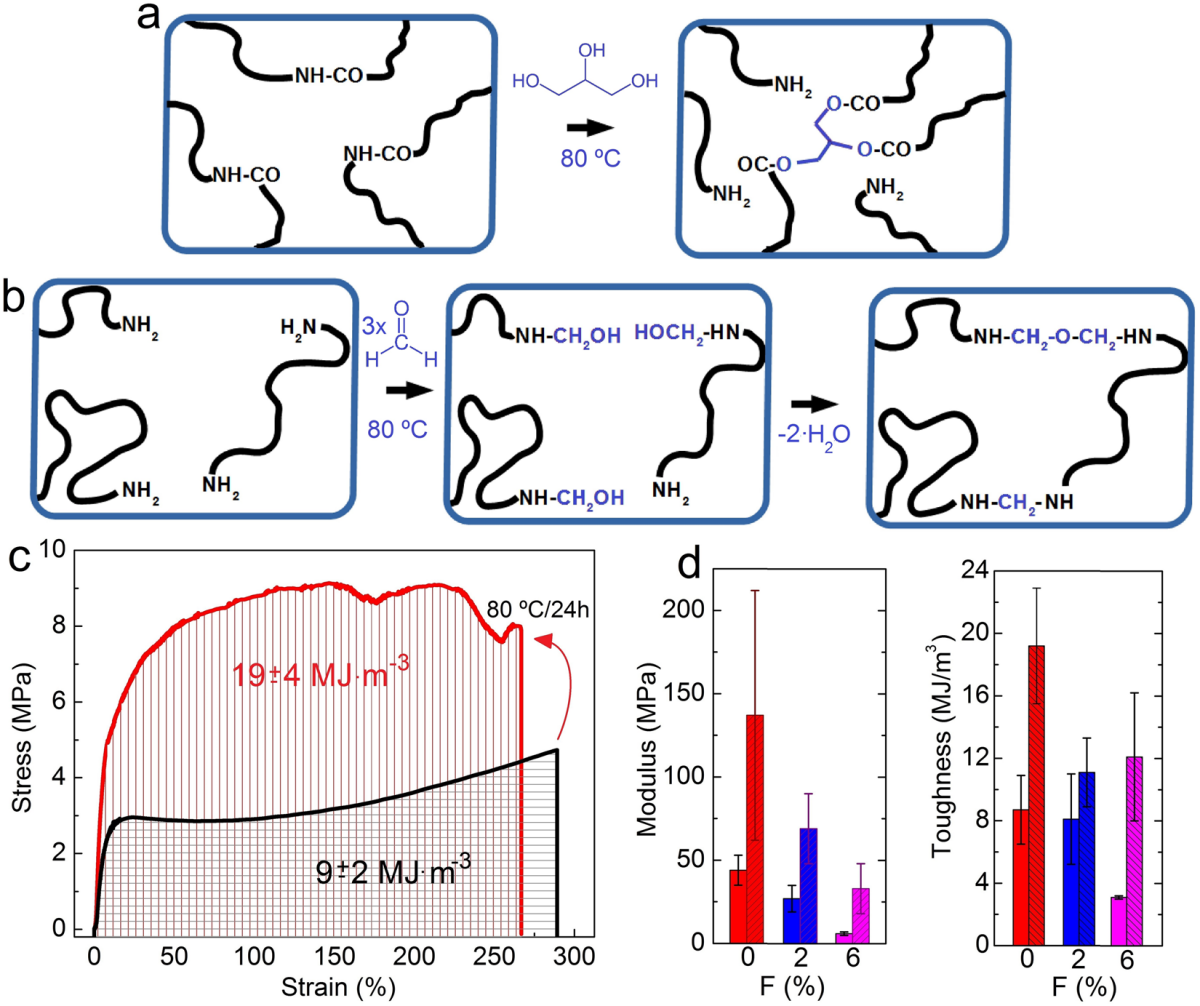
Scheme of the proposed mechanisms of keratin cross-linking by the action of (**a**) glycerol and (**b**) formaldehyde. (**c**) Representative stress-strain curves of 28 wt%Gly bioplastic before and after thermal cross-linking treatment at 80 °C. (**d**) Elastic moduli and toughness of bioplastics with 28 wt% Gly formed from solutions with 0, 2 and 6 wt% of formaldehyde. Data for cross-linked materials are dashed.

Plastics containing 28% of glycerol were thermally cross-linked and the effect of formaldehyde addition was analysed. The action of glycerol is not clear at this stage but it is plausible that the cross-linking ability is based on the esterification of amide function by hydroxyls from glycerol and therefore promoting network formation between three different keratin macromolecules (Fig. 4a). Formaldehyde is known to act as a protein cross-linker which has the ability to react with two different secondary or primary amino groups of a protein structure favouring its chain extension and cross-linking trough the formation bridges based on functional groups such as ether, methylene or imine (Fig. 4b)^64,65^. Other plausible cross-linking reactions comprise the formation of sulphonyl formamides upon the reaction of sulfonic acids with terminal amides in the presence of aldehyde^66^. The cross-linking is demonstrated by solubility tests in basic aqueous solutions in which it is observed that after the thermal cross-linking only the pure keratin film remains soluble. The glycerol and formaldehyde convert the material insoluble in water leading to hydrogels (Fig. S3).

Figure 4c shows representative stress strain curves of keratin films containing 28% glycerol before and after thermal treatment at 80 °C for 24 h. Figure 4d show data of elastic moduli and toughness of materials with 28% glycerol prepared from film forming solutions containing 0, ~2 and 6% of formaldehyde, respectively, before and after treatment at 80 °C. It can be seen that the effect of the chemical cross-linking is not revealed before the thermal treatment at 80 °C. On the contrarily, the presence of free formaldehyde might have certain plasticizing effect as evidenced by the lower moduli and tensile strengths exhibited by materials with increased amount of formaldehyde. As it can be seen the thermal treatment leads to unique materials characterized by their unprecedented toughness for this type of protein films. The material cross-linked only with glycerol exhibited a comparable modulus to that of the bioplastic with 16 wt% glycerol, but with a deformation about five times higher and a significant superior tensile strength. Thermal cross-linking of plasticized keratin films, thus, lead to materials with unprecedented toughness. Films with 28 %Gly and cross-linked at 80 °C endow a toughness of 19 ± 4 MJ·m^−1^.

**Figure 5:**
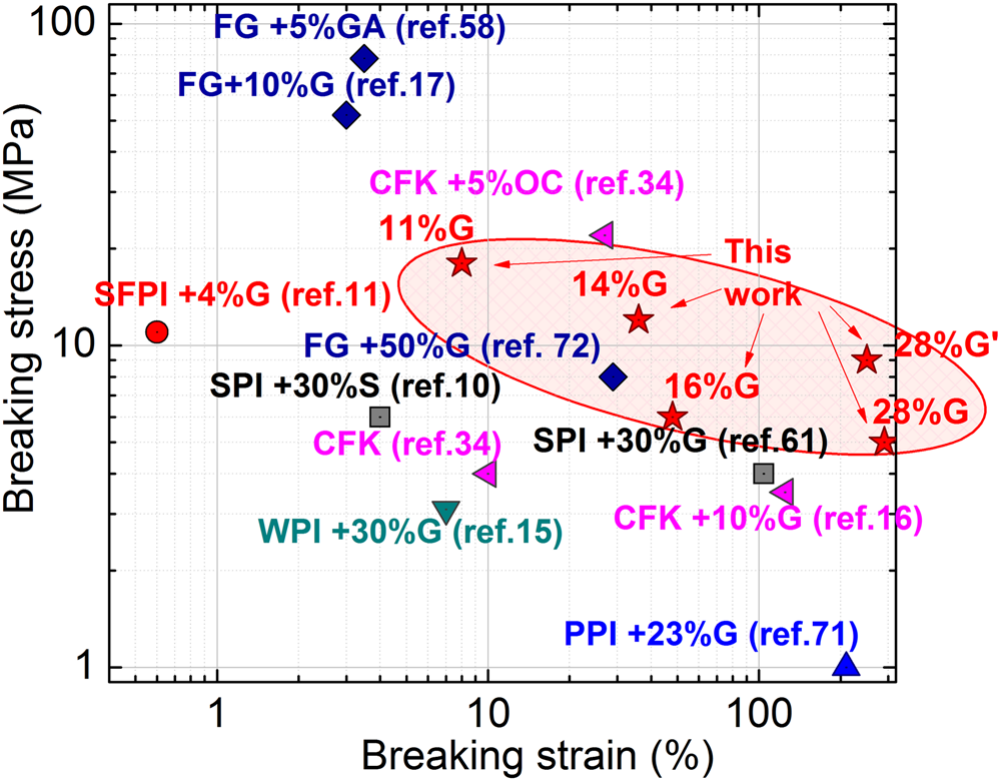
Comparison of the stress and strain at break of the films obtained here with results reported by other authors for other protein based bioplastics. G refers to glycerol and G′ refers to the cross-linked bioplastic.

In general the toughness values of thermally cross-linked bioplastics are much higher than those obtained by other authors for protein films. For example, recently Song *et al*. obtained bioplastics from chicken feather keratins (CFK) reinforced with dialdehyde cellulose nanocrystals (DCNC)^34^. They obtained the best performance with a 5 wt% of DCNC exhibiting a strength of 22 MPa and a deformation at break of 30%, resulting in a toughness of about 5.4 MJ·m^−3^. In Fig. 5 mechanical data of bioplastic films analysed here are summarized and presented along with data obtained in the literature. It can be seen that properties of materials analysed here rely on the tough side of the chart, in which high deformations and good strengths are exhibited. For example, the bioplastics developed here exhibit a higher strength than that reported for CFK. Only CFK cross-linked with 5 wt% of DCNC presented slightly higher strengths. On contrast while the plasticized wool keratin films with 28 wt% glycerol and thermally cross-linked with formaldehyde shows half about half of the strength of the DCNC cross-linked CFK, it presents almost an order of magnitude higher deformation at break, therefore endowing a much higher toughness. Mechanical properties and, particularlly, toughness of other films based on sunflower protein isolate (SFPI), soy protein isolate (SPI), whey protein isolate (WPI), fish gelatin (FG) or pea protein isolate (PPI) have also been reported to be generally inferior.

### Biodegradation and analysis of wool derived bioplastics life cycle

#### Biodegradation

**Figure 6:**
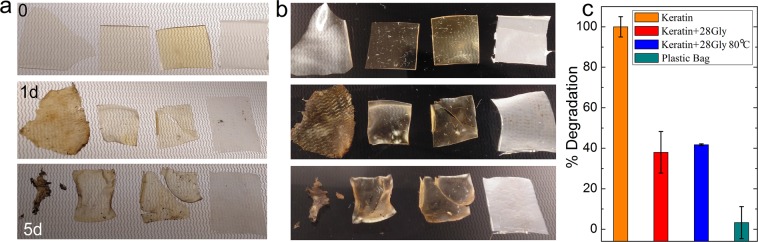
Bioplastic films as observed by (**a**) transmitted light and (**b**) between crossed-polarizers after different times composting conditions (0, 1 and 5 days). From left to right a pure keratin film, keratin +28 wt% glycerol, keratin +28 wt% glycerol cross-linked at 80 °C, and a commercial starch based compostable plastic bag (*PlasBel*). (**c**) Weight of degraded bioplastic after 5 days at composting conditions for the four representative materials.

Pure keratin films under composting conditions degraded completely within 5 days (Fig. 6). At the same period of time films of a commercial starch based bag only degraded to a minimum extent. Films with glycerol, physically mixed and thermally cross-linked exhibited an intermediate behaviour with around 40 wt% degradation at the same period of time. The effect of glycerol on biodegradation might be related to partial cross-linking reactions between keratin macromolecules. As observed between cross-polarizers plasticized films appear more amorphous than pristine keratin films. On the other hand commercial starch based bag appears as a highly birefringent material even after composting conditions. This can be ascribed to the high degree of crystallisation of the hydrophobic phase of which these bags are partially composed. The pure keratin film appears to lose its birefringence upon degradation due to its high degree of hydration. On the contrarily films plasticized and cross-linked with glycerol increase its birefringence upon degradation. This can be explained by the nucleation effect of cross-links over the rest of macromolecules upon loss of free molecules like water, non-cross-linked glycerol and short migrating oligopeptides. As present experiments of degradation at soil can be explained in terms of water hydration and its solubilising effect, it is envisioned that similar trend would be observed *in-vivo*, at the aim of exploring these materials as supports for living tissue regeneration.

#### Life cycle analysis

Breeding lambs or chicken for the solely purpose of producing keratin based bioplastics from their wool and feathers would not be beneficial in terms of sustainability for the extension of the land use. It is for this reason that materials such as leather endow a very high energy finger-print^25^. In a parallel way Life Cycle Analysis (LCA) of plant protein bioplastics, such as those derived from soy have cast doubts on its present sustainability due to the extent of land and fertilizers used for its production^67^. But at present, due to bad quality of certain wools and feathers they are rendered as residues. Sheep is a source of milk, cheese and meat and wool is a side-product. Therefore when performing a LCA this consideration should be taken into account together with the benefits of an appropriate management of these residues and of a partial substitution of currently available plastics derived from oil. In addition, LCA analysis of other protein based films have pointed out that biodegradable plastics might have a high positive impact at the end of their Life Cycle in a composting scenario, due to the fast integration into the soil by microorganisms and because of the reduction of the amount of waste sent to other treatments such as incineration, avoiding emissions associated to these treatments^17,67^.

## Conclusions

Wool residues can be considered as an attractive source of macromolecules for small productions of bioplastics with specific applications. The wool keratins can be derived into water soluble proteins with high molecular weight by means of simple chemical treatments and can be turned into films with plastic properties by using different ratios of plasticizers. The use of amphiphilic plasticizers such as SDS leads to more hydrophobic material but with less plasticizing effect. The materials show good transparency, UV barrier properties, thermal stability in the range of 50–200 °C, sensitivity to humidity and mechanical properties comparable with the best reported protein bases plastics. Specifically, when cross-linked, the plasticised bioplastic exhibited superb fracture energy absorption, as determined by their high intrinsic toughness. These protein based films might find applications in a diverse range of fields such as regenerative medicine, bioplastics, coatings or packaging.

## Materials and Methods

### Materials

Wool from *lacha* black face (Spanish: *Lacha caranegra*; Basque: *Latxa mutturbeltz*; French: *Manech*; Latin: *Ovis aries*) was kindly provided by *Quesería-Etxetxipia-Gaztegieta*, a cheese farm in Elizondo, middle-north Spain. Fibres consisted on ~30 cm long strands in which tips were slightly more yellowish than the stems.

### Keratin extraction

Wool fibres were washed twice into a washing machine with solid surfactant. Two more times were washed only with water. The fibres were then dried at 25 °C for several days. The keratin extraction with H_2_O_2_ as bisulfide splitting agent was carried out as explained elsewhere^36^. Briefly, wool (75 g) was cut into ~2 cm long fibres and suspended into 2 M H_2_O_2_ aqueous solution (1.2 L). To aid oxidation, of NaClO (75 mL) at % were added, although this might reduce extraction yield due to the possible formation of cross-links such lisinoalanins, lanthionines or sultones^36,68,69^. The pH of this suspension was raised to pH ~10, the solution volume completed to 1.5 L with ultrapure deionized water (Wasserlab, Pamplona, Spain) and the temperature taken to 50 °C. The reaction was hold for 2 h (see Fig. S1a), when additional 2 L ultra-pure water were added as reaction quenchers. This solution was filtrated through filter paper and about 81% of yield was obtained, as measured from the solid residues remaining in the filter. An aliquot of 15 mL of the filtrate was titrated by adding drop-wide HCl 3 N and by measuring the pH with an electronic pH-meter. The pH of the filtrate was dropped by adding drops of HCl (3 N) until pH ~ 4, when keratin precipitated as a white polymer (Fig. S1b,c). The precipitate was washed with an isopropanol/water mixture and stored into the fridge. One oxidative extraction with H_2_O_2_ (0.5 N) was carried out to compare the molecular distribution weight obtained with a reductive extraction with thioglicolic acid (HS-CH_2_-COOH, 0.5N) as bisulfide splitting agent.

### Determination of the molecular weight distribution (SDS-PAGE)

The precipitates were inserted into cellophane tubes (Viscofán S.A, Spain) and dialyzed against ∼16 MΩ ultrapure water (Wasserlab, Pamplona, Spain) 3 days, changing the water regularly. Molecular weight of keratins was determined by sodium dodecyl sulphate (SDS) polyacrylamide gel electrophoresis (PAGE). The gels were made up of three distinct fractions in order to have a good resolution of the high and low molecular weight keratin fractions. The gel fractions contained 4, 10 and 18 wt% of acrilamide/bisacrilamide (Acril/Bis) and were buffered with Tris-HCl, 0.375 M at pH = 8.8. Tris-HCl stands from tris(hydroximethyl)aminomethane (Panreac, Spain) neutralized with HCl up to the indicated pH. Keratin samples (~3 mg) were dispersed into a buffer based on Tris-HCl 20 mM, pH = 8.8; 2% SDS, urea 3.6 M, 15% de glycerol and 5% de β-mercaptoethanol (β-M, Panreac) up to a concentration of ∼4 mg·mL^−1^. Bromophenol blue was pipetted to a concentration of ∼0.05 wt% and the samples incubated into 2 mL centrifuge (*eppendorfs*) tubes at 97 °C for 3 min s. Then, samples were sonicated into bath for 10 min and centrifuged for ∼10 s. A volume of 7 μL of sample and protein marker (*Protein Marker VI*, 10–245 KDa, Panreac, Spain) was drop into the bottom of the wells. The electrophoresis run at 200–240 V into a Miniprotean, BioRad with a Tris-glycine-SDS (0.375 M, pH = 8.8) buffer, until the bromophenol reached the bottom of the third gel. The gels were tinted with a 5 wt% Coomassie Blue solution in a mixture of methanol and acetic acid. The gels were revealed by washing with a mixture of methanol and acetic acid, stored in plastic bags into the fridge with part of the revealing solvent and scanned using a HP-*Scanjet-*5590 scanner and analysed using *ImageJ* free software.

### Preparation of keratin bioplastic films

The keratin precipitate was concentrated in a mixture of isopropanol/water (~50:50) to a concentration of 63 g·L^−1^. Keratin was dissolved by drop-wise addition of concentrated NaOH up to pH ~ 10. Aliquots (15 mL, ~0.95 g of keratin) of this solution were mixed with different volumes of glycerol (50%vol./H_2_O solution) and 10 wt% SDS, and were sonicated in bath for ~10 s before being cast into silicon moulds. These were placed into a lab hood and the water evaporated with a constant air flow at ambient temperature (24 ± 2 °C). Blank samples were cast with no additives and once dried were weighed to determine keratin concentration in the stem solutions. With this methodology bioplastic films containing 4, 6, 11, 14 and 16 wt% of glycerol and 6, 11 and 16 wt% of SDS were obtained.

### Elemental analysis

Atomic composition of wool fibroin and keratin films was performed analysing ~5 mg of dried samples in a TruSpec-Micro (LECO) analyser.

### Fourier transformed infrared spectroscopy (FTIR)

Keratin based bioplastics were analysed by attenuated-total-reflection (ATR) FTIR spectroscopy into a *Jasco* FTIR spectrophotometer recording 25 scans with 4 cm^−1^ resolution in the range of 4000–400 cm^−1^. A spectra of washed wool fibres were recorded for comparison.

### Optical characterization by UV-Vis spectroscopy

Ultraviolet and visible light spectra (UV-Vis) were recorded in the wavelength range of 800–200 nm using a *DH-mini* UV-Vis-NIR light source from *Ocean Optics* instruments. Films were placed across the beam and spectra was recorded. Films opacity was defined as the absorbance at 600 nm divided by the film thickness (as measured with a digital micrometer).

### Thermogravimetric analysis (TGA)

Thermogravimetric analysis (HI-RES 2950, *TA Instruments*) of washed wool and bioplastic films were performed in the temperature range of 25–600 °C at a heating rate of 10 °C·min^−1^ in an air atmosphere with a constant 60 mL·min^−1^ air flow. The samples weights were of about 2.5 mg.

### Dynamic-mechanical thermal analysis (DMTA)

Tests were carried out in a DMA-Eplexor, Gabo Qualimeter, dynamic analyser. Specimens were rectangular ~5 × 0.15 mm^2^ and the initial cross-head distance of 2.5 cm. The initial contact force was of 0.5 N, the dynamic load of 0.03%, the heating rate of 3 °C·min^−1^, and the scanning frequency of 1 Hz. The temperature range analysed was from –100 °C to 200 °C.

### Impact of plasticizer nature in H_2_O absorption kinetics

Three differentiated pieces with weight in the range of 20–60 mg of films of pure keratin and bioplastics with 16% of SDS and Glycerol were dried to a constant weight at 90 °C and applied vacuum. They were weighed again (*m*_0_) and set into individual wells of a plastic blister. This was inserted into a zip plastic bag of 10 cm × 15 cm (maximum volume of ~315 cm^3^), containing a saturated NaCl solution (65 ± 3%RH, *T* = 24 ± 3 °C). The RH was checked using an electronic pocket Humidity Meter (HDT-305E) provided with a tip probe. The RH dropped to ambient condition when the zip was opened but recovered the original value in about 10 min once the zip was closed again. Samples were withdrawn from the bag and weighed at different periods of time (*m*_t_). The same experiment was repeated with a three bunches (0.2–0.9 g) of washed and dried wool. The absorbed H_2_O was expressed as:

2 $${\rm{Absorbed}}\,{{\rm{H}}}_{2}{\rm{O}}( \% )=100\cdot ({m}_{t}-{m}_{0})/{m}_{0}$$

### Impact of RH on absorbed H_2_O in equilibrium

The maximum absorbed water into films with 14 wt% glycerol was determined under environments with different humidity at a temperature of 24 ± 3 °C. Three dried pieces were inserted into zip plastic bags containing saturated salts solutions achieving different atmosphere with different RH (LiCl, 16 ± 2 %RH; desiccator, 30 ± 3%RH; ambient, 53 ± 2%RH; NaCl, 65 ± 3%RH; KCl, 70 ± 3%RH; Na_2_CO_3_, 79 ± 3%RH).

### Tensile testing

Samples for tensile testing were conditioned for a minimum of 48 h at 24 ± 3 °C into a desiccator (RH ~30 ± 3%). In addition, samples of bioplastic film with 14%wt glycerol were conditioned in other environments, as described above, in order to assess the impact of water plasticizing effect. The specimen dimensions were of ~3.5 × 0.5 mm^2^ cross section. The initial cross-head distance was of 8 mm. Tensile tests were performed in a Shimadzu tensile tester equipped with a 200 N loading cell. The cross-head speed was set to 5 °C·min^−1^. A minimum of three specimens where measured per sample.

### Biodegradation experiments

Representative samples conditioned into a desiccator (~40 %RH) were cut into ~1.5 × 2 cm^2^ pieces, weighed and immersed into commercial compost derived soil (Abonos Naturales, *Hnos*. *Aguado S*.*L*), with 37.3% organic matter, 13.9% organic carbon, 0.8% organic nitrogen, 1.3% total nitrogen and a pH of 8.2. The soil (20 Kg) was mixed with of deionized water (8 Kg) into a 25 cm deep plastic bag with a surface of 30 × 50 cm^2^. Samples were submerged at about 5 cm from the surface and water was spread on top regularly to maintain the water content constant. Samples were withdrawn at differ times, conditioned into a desiccator (~40 %RH) and weighed. Experiments were performed by triplicate.

## Supplementary information

Supplementary information
