# Supplementary material for: Tough and Functional Cross-linked Bioplastics from Sheep Wool Keratin
Source: Sci Rep. 2019 Oct 15;9:14810. doi: 10.1038/s41598-019-51393-5 (PMC6794297; doi:10.1038/s41598-019-51393-5)
Supplement: Supplementary file 1 — Supplementary information [file 41598_2019_51393_MOESM1_ESM.docx]

**Supplementary Material**

Tough and Functional Cross-linked Bioplastics from Sheep Wool Keratin

*Borja Fernández-d'Arlas*

Institute for Advanced Materials (INAMAT), *Universidad Pública de Navarra* (UPNA), *Centro Jerónimo de Ayanz*, Pamplona, España (Spain). *contact: borja.fernandezdarlas@unavarra.es

CONTENT:

- **FIGURE S1:** Wool solubilisation and keratin precipitation.
- **FIGURE S2:** Keratin characterization and molecular weight determination by SDS-PAGE
- **FIGURE S3:** Solubilisation tests of keratin plastics after thermal treatment at 80 ºC.

**FIGURE S1**


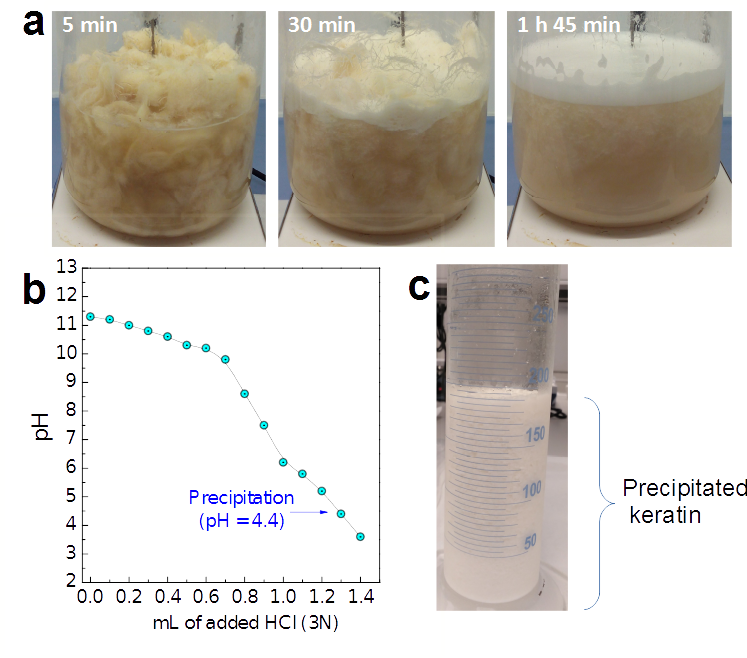


**Figure S1**. a) Wool keratin extraction vessel at different period of times. b) Titration curve of a 15 mL aliquot of the filtrate. c) Wool keratin precipitated at pH ~ 3.

**FIGURE S2**

**
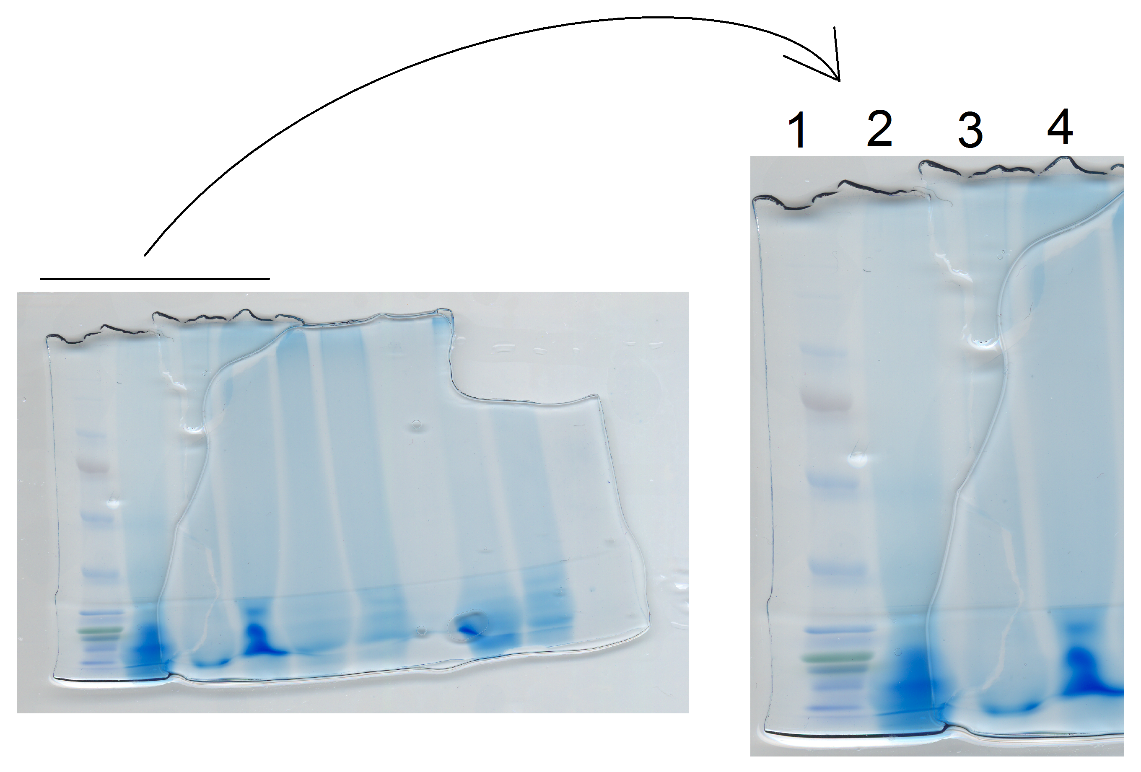
**

The upper gel and the detail to the right comprises a polyacrylamide gel. The numbers indicate: 1) protein marker, 2) wool keratin extracted with thioglycolate analyzed in a buffer containing β-mercaptoethanol, 3) wool keratin extracted with thioglycolate analyzed in a buffer without β-mercaptoethanol (protein stacked in the concentrating gel) and 4) wool keratin extracted with H_2_O_2_ and analyzed with β-mercaptoethanol. Analysis in detail is provided below.


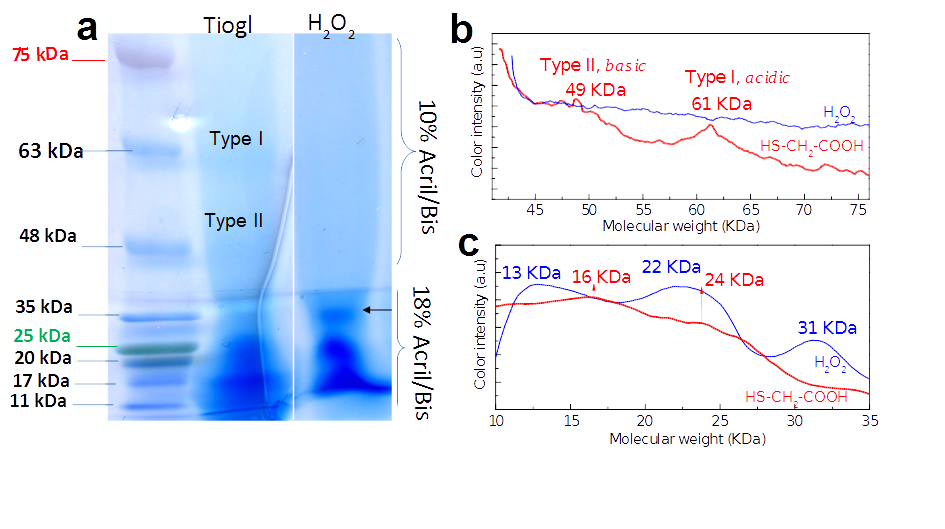
**Figure S2**. a) SDS-PAGE Electropherograms of the protein marker, keratin extracted with thioglycolid acid and H_2_O_2_. Color intensity profile against molecular weight distribution in the a) 10% Acril/Bis gel fraction and c) 18% Acril/Bis gel fraction. The blots were cropped horizontally in order to provide a direct comparison between the extraction methods


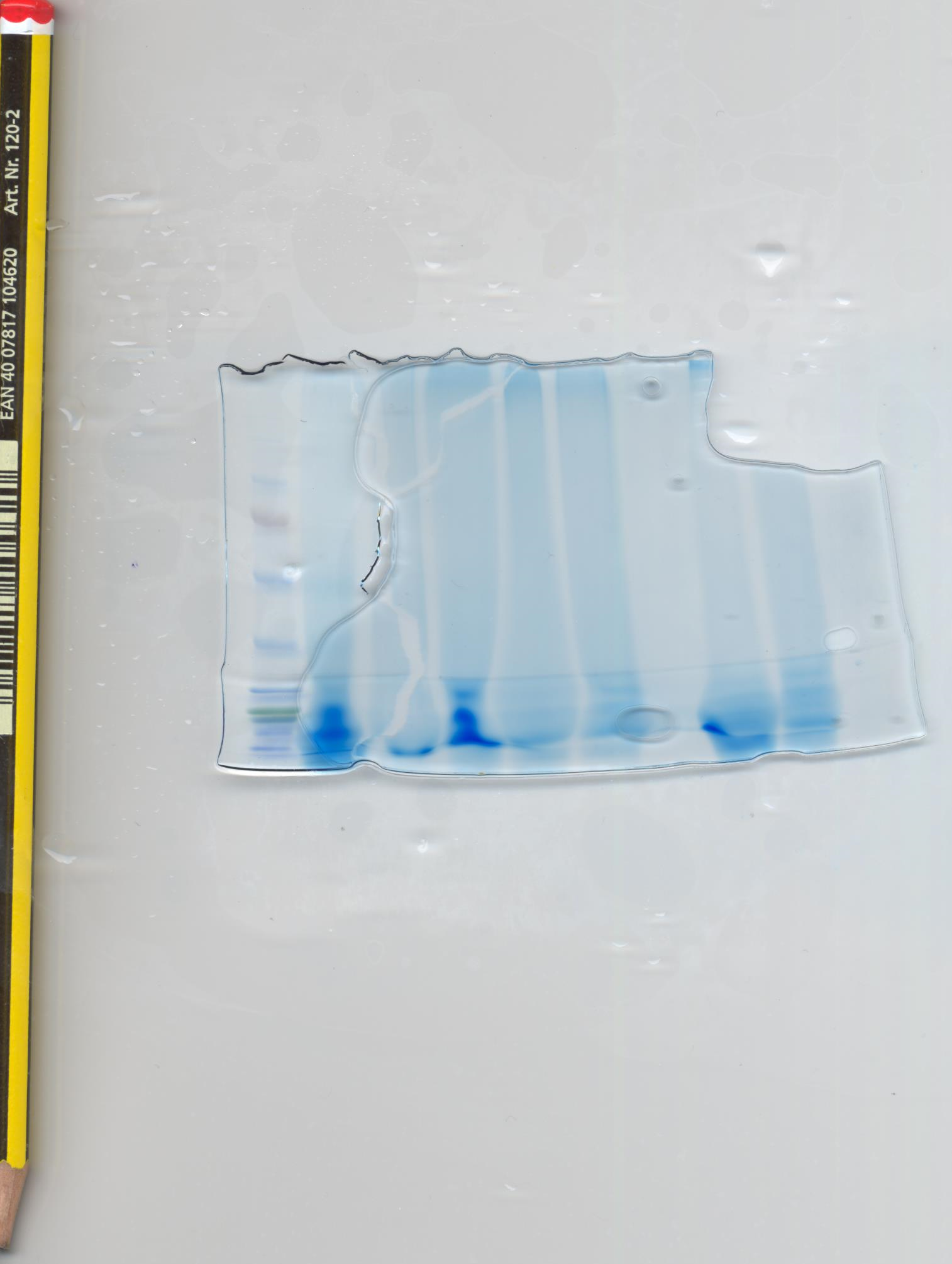


The upper figure corresponds to a different scan of the same gel shown above.

**FIGURE S3**


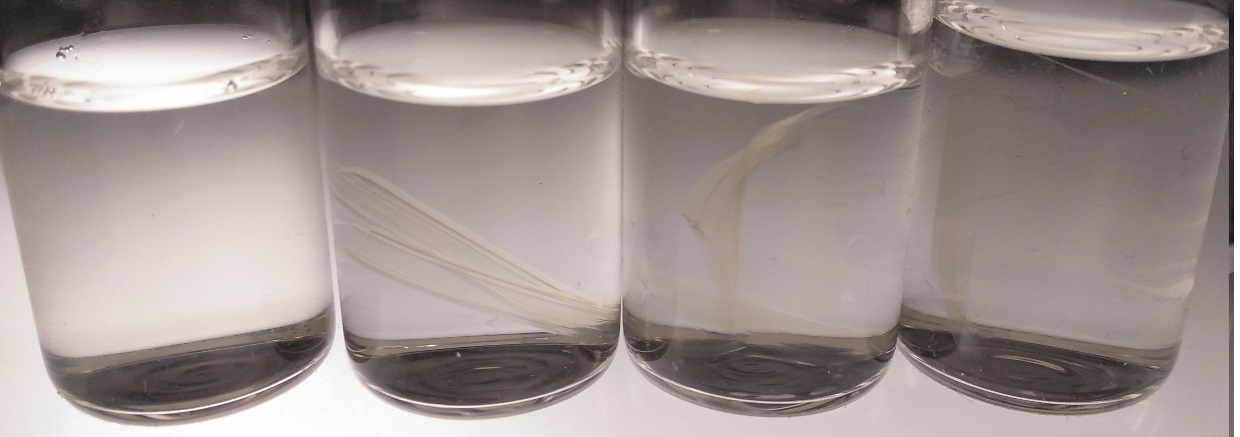


**Figure S3**. Solubility tests of wool keratin samples after thermal treatment at 80ºC for 24h. From left to right, pure keratin samples (completely soluble) and samples with 28 wt% glycerol cast from solution with 0, 2 and 6 % of formaldehyde, respectively (insoluble).
